# Apocynum Cannabinum in Heart Disease

**Published:** 1894-12

**Authors:** 


Apocynum Cannabinum in Heart Disease.—Dr. Glinski, after having proved by experiments on cold-blooded and warm-blooded animals that the root of the apocynum cannabium contains a strong poison which in large doses paralyzes the heart, and when given in small quantities retards and strengthens its beats, decided to take it himself, as he is suffering from hypertrophy of the left ventricle, with intercurrent attacks of dilatation of the organ, mitral murmur, dyspnœa, etc. (*The British Medical Journal.*) The dose was fifteen drops of the fluid extract three times a day. As he found that all his symptoms disappeared in two days, he gave it also to other patients in the same quantity in cases of palpitation, disturbed compensation, in which strophanthus, and adonis vernalis had failed and digitalis seemed contraindicated. He gives a full account of some of his cases, and summarizes his experience in the following conclusions: 1. The action of the root of apocynum cannabinum is similar to that of digitalis, without being cumulative. 2. In cases of dilatation, the fluid extract rapidly diminishes the area of dulness. 3. It increases the daily amount of urine, stops the palpitation and promotes the absorption of transudations. 4. With the exception of increased pulsation of the arteries of the head, it has no bad secondary effects. It was used either in the form of a decoction (ʒ j. to ℥ viij), 3 to 4 tablespoonfuls a day, or tincture (1 in 10), 5 to 10 minims, three to four times daily, or fluid extract in doses of 10 minims to half a teaspoonful three times daily.—*Medical Record.*

